# Bullous pemphigoid and mucous membrane pemphigoid humoral responses differ in reactivity towards BP180 midportion and BP230

**DOI:** 10.3389/fimmu.2024.1494294

**Published:** 2024-11-29

**Authors:** Feliciana Mariotti, Anna Pira, Naomi De Luca, Anna Rita Giampetruzzi, Filomena Russo, Amilcare Cerri, Giulia Gasparini, Emanuele Cozzani, Angelo V. Marzano, Emiliano Antiga, Marzia Caproni, Pietro Quaglino, Marco Carrozzo, Biagio Didona, Giovanni Di Zenzo

**Affiliations:** ^1^ Molecular and Cell Biology Laboratory, Istituto Dermopatico dell’Immacolata (IDI)-IRCCS, Rome, Italy; ^2^ Dermatology Unit, Istituto Dermopatico dell’Immacolata (IDI)-IRCCS, Rome, Italy; ^3^ Dermatological Clinic, Department of Health Sciences, University of Milan, AO Santi Paolo e Carlo, Milan, Italy; ^4^ Section of Dermatology, Department of Health Sciences (DISSAL), University of Genoa, Genoa, Italy; ^5^ Dermatology Unit, IRCCS Ospedale Policlinico San Martino, Genoa, Italy; ^6^ Dermatology Unit, Fondazione IRCCS Ca’ Granda Ospedale Maggiore Policlinico, Milan, Italy; ^7^ Department of Pathophysiology and Transplantation, Università Degli Studi di Milano, Milan, Italy; ^8^ Section of Dermatology, Department of Health Sciences, University of Florence, Florence, Italy; ^9^ Immunopathology and Rare Skin Diseases Unit, Section of Dermatology, Department of Health Sciences, Azienda Unità Sanitaria Locale Toscana Centro, European Reference Network-Skin member, University of Florence, Florence, Italy; ^10^ Dermatologic Clinic, Department of Medical Sciences, University of Turin, Turin, Italy; ^11^ Oral Medicine Department, School of Dental Sciences, Newcastle University, Newcastle upon Tyne, United Kingdom; ^12^ Rare Diseases Unit, Istituto Dermopatico dell’Immacolata (IDI)-IRCCS, Rome, Italy

**Keywords:** bullous pemphigoid, mucous membrane pemphigoid, autoantibody, humoral response, epitope, autoantigen

## Abstract

**Background:**

Bullous pemphigoid (BP) and mucous membrane pemphigoid (MMP) are rare autoimmune blistering disorders characterized by autoantibodies (autoAbs) targeting dermo-epidermal junction components such as BP180 and BP230. The differential diagnosis, based on both the time of appearance and the extension of cutaneous and/or mucosal lesions, is crucial to distinguish these diseases for improving therapy outcomes and delineating the correct prognosis; however, in some cases, it can be challenging. In addition, negative results obtained by commercially available enzyme-linked immunosorbent assays (ELISAs) with BP and MMP sera, especially from patients with ocular involvement, often delay diagnosis and treatment, leading to a greater risk of poor outcomes.

**Objectives:**

Our aim was to find potentially different reactivity profiles in BP and MMP and improve available approaches for diagnosis with focus on ocular MMP.

**Methods:**

Two cohorts of 90 BP and 90 MMP, recruited from different Italian clinical centers, were characterized also employing a novel ELISA based on the BP180 extracellular domain (ECD-BP180).

**Results:**

Immunoglobulin G (IgG) reactivity to BP180 and BP230 in MMP sera was significantly reduced in comparison with BP, mostly affecting BP230 and E-1080 (53% and 36% in BP vs. 11% and 3% in MMP, respectively, *p* < 0.0001). The combined sensitivity of BP180-NC16A and ECD-BP180 ELISAs was greater compared to BP180-NC16A and BP230 ELISAs both in BP (97% and 92%, respectively) and in MMP (42% and 31%, respectively). The present study shows that MMP patients with ocular involvement rarely reacted to BP180 by IgG in contrast with patients with oral and/or cutaneous involvement (*p* = 0.0245 and *p* = 0.0377, respectively), suggesting that an oral and/or cutaneous MMP positive to BP180 hardly evolves to ocular MMP. Of note, one-third of ocular MMP showed immunoglobulin A (IgA) reactivity to ECD-BP180 by immunoblotting.

**Conclusions:**

The present study provides several hints to perform a correct and timely diagnosis in BP and MMP, which is crucial for improving therapy outcomes and delineating the correct prognosis.

## Introduction

Pemphigoid diseases are rare autoimmune blistering disorders characterized by the binding of autoantibodies (autoAbs) to several components of the dermal–epidermal junction. Tense blisters and erosions, affecting skin and/or mucous membranes, are clinical hallmarks of pemphigoids. Moreover, linear deposits of immunoglobulin G (IgG), and less often immunoglobulin A (IgA), and/or complement fragment 3 (C3) are detected in direct immunofluorescence (DIF) of perilesional biopsies.

Bullous pemphigoid (BP) and mucous membrane pemphigoid (MMP) were identified as the most prevalent pemphigoid disorders (1). BP mainly affects the elderly, arising in people aged between 70 and 80 years (2, 3). Two hemidesmosomal proteins, the intracellular BPAG1/BP230 and the type II transmembrane BPAG2/BP180, are targeted by autoAbs (4, 5). BP180 is the main antigen in BP and its immunodominant region, the non-collagenous 16A domain (NC16A), accounts for nearly 90% and 65% of IgG and IgA reactivity, respectively (6–8). However, other epitopes in the extracellular domain of BP180 (ECD-BP180), spanning from the midportion to the C-terminal, are also bound by autoAbs (6, 9, 10).

MMP is a heterogeneous subepidermal bullous disorder with a predominant or exclusive mucosal involvement, sometimes with limited skin lesions that can evolve into atrophic scars. Lesions may occur in one site or in multiple sites, with the oral cavity being the most frequently affected area (11–15). Besides BP180 and BP230, MMP sera react with other structural components of the basement membrane zone underlying the epidermis and epithelia, namely, laminin 332, α6β4 integrin, and type VII collagen (15–18). Compared to BP, the titer of circulating autoAbs is lower in MMP sera (19, 20). As for IgG, reactivity to NC16A is present in a percentage of patients ranging from 33% to 53% (16, 21), and other epitopes in the C-terminus of BP180 are also recognized in 16-53% of patients (20, 22). IgA reactivity was also described, and variable frequencies (0%–28% and 4%–37% to NC16A and C-terminus, respectively) were reported (23).

The differential diagnosis between BP and MMP, based on clinical criteria such as the time of appearance and the extension of cutaneous and/or mucosal lesions, is crucial for improving therapy outcomes and delineating the correct prognosis; however, in some patients, it can be challenging.

To characterize the humoral response and find potentially different serological reactivity, we clinically and immunologically characterized two cohorts of 90 BP and 90 MMP. Moreover, to improve the diagnostic performance of commercial enzyme-linked immunosorbent assay (ELISA) kits, IgG and IgA reactivity against the recombinant form of the extracellular domain of BP180 was assessed.

## Materials and methods

### Patients

A total of 90 BP and 90 MMP patients were retrospectively and prospectively recruited in five Italian clinical centers (Rome, Genoa, Florence, Turin, and Milan) and characterized. The diagnosis of BP and MMP was established as described in published guidelines (24, 25). The differential diagnosis between BP and MMP was based on the time of appearance and the extension of cutaneous and/or mucosal lesions (24, 25). Indirect immunofluorescence (IIF) on salt-split skin sections was performed as reported in Calabresi et al. (20). Clinical and immunological characteristics are summarized in **Tables 1**, **2**. This study was carried out with the approval of the IDI-IRCCS Ethics Committee. All biological samples were obtained after patients’ informed consent, in adherence to the Helsinki Principles.

**Table 1:** Demographic, clinical, and immunological characteristics of 90 bullous pemphigoid patients.

	*n* (%)	
**Mean age (years) (± SD) (*N* = 84)**	76.8 (± 12.3)	
**Women (*N* = 84)**	41 (48.8)	
**Mucosal involvement (*N* = 83)**	20 (24.1)	
** Oral**	11 (13.3)	
** Genital**	1 (1.2)	
** Multiple mucosal sites**	4 (4.8)	
Unknown*	4 (4.8)	
**IIF-sss (epidermal side, IgG) (*N* = 88)**	87 (98.9)	**Mean IgG titer** **(U/mL or PIV)**
**IgG anti-BP180-NC16A by ELISA (*N* = 90)**	79 (87.8)	119.3
**IgG anti-BP230 by ELISA (*N* = 90)**	48 (53.3)	64.3
**IgG anti-E-1080 by ELISA (*N* = 89)**	32 (36.0)	101.0
**IgG anti-E-1331 by ELISA (*N* = 89)**	35 (39.3)	40.6
**IgG anti-ECD-BP180 by ELISA (*N* = 90)**	71 (78.9)	79.8
**IgA anti-ECD-BP180 by IB (*N* = 55)**	30 (54.5)	
**Combined ELISAs (BP180-NC16A, BP230,** **E-1080, and ECD-BP180)**	90 (100)	

E-1080 and E-1331, midportion (AA 1,080–1,107) and C-terminal region (AA 1,331–1,404) of the extracellular domain of BP180; ECD-BP180, ectodomain of BP180 (AA 490–1,497). *Data on mucosal involvement are known in 16 out of 20 bullous pemphigoid patients. Mean IgG titer was calculated on positive sera. PIV, pemphigoid index units; U, units; IB, immunoblotting; IIF-sss, indirect immunofluorescence on salt-split skin.

**Table 2:** Demographic, clinical and immunological characteristics of 90 mucous membrane pemphigoid patients.

	*n* (%)	
**Mean age (years) (± SD) (*N* = 53)**	67.5 ( ± 13.3)	
**Women (*N* = 89)**	59 (66.3)	
**Mucosal involvement (*N* = 86^A^)**	86 (100.0)	
**Oral**	42 (48.8)	
**Oral and cutaneous**	5 (5.8)	
**Ocular**	13 (15.1)	
**Ocular and cutaneous**	4 (4.7)	
**Multiple mucosal sites**	3 (3.5)	
**Multiple mucosal sites and cutaneous**	19 (22.1)	
**IIF-sss, IgG (*N* = 90)**		
**Epidermal side **	31 (34.4)	
**Dermal side**	3 (3.3)	
**IIF-sss, IgA (*N* = 77)**		
**Epidermal side**	18 (23.4)	
**Dermal side**	7 (9.1)	**Mean IgG titer** **(U/mL or PIV)**
**IgG anti-BP180-NC16A by ELISA (*N* = 90)**	25 (27.8)	28.0
**IgG anti-BP230 by ELISA (*N* = 90)**	10 (11.1)	26.3
**IgG anti-E-1080 by ELISA (*N* = 90)**	3 (3.3)	45.6
**IgG anti-E-1331 by ELISA (*N* = 90)**	6 (6.7)	54.0
**IgG anti-ECD-BP180 by ELISA (*N* = 90)**	28 (31.1)	42.3
**IgA anti-ECD-BP180 by IB (*N* = 76)**	30 (39.5)	
**IgG anti CollVII by ELISA (*N* = 68)**	3 (4.6)	
**IgG anti-Lam332 by IB (*N* = 75)**	11 (14.7)	
**Combined immunological assays** **(all the above assays except E-1331 ELISA^B^)**	75 (83.3)	

E-1080 and E-1331, midportion (AA 1,080–1,107) and C-terminal region (AA 1,331–1,404) of the extracellular domain of BP180; ECD-BP180, ectodomain of BP180 (AA 490–1,497). ^A^Data on mucosal involvement are known in 86 out of 90 mucous membrane pemphigoid patients. ^B^E-1331 ELISA reactivity does not improve the global sensitivity. Mean IgG titer was calculated on positive sera. PIV, pemphigoid index units; U, units; IB, immunoblotting; IIF-sss, indirect immunofluorescence on salt-split skin.

### ECD-BP180 cloning and production

**Figure 1:**
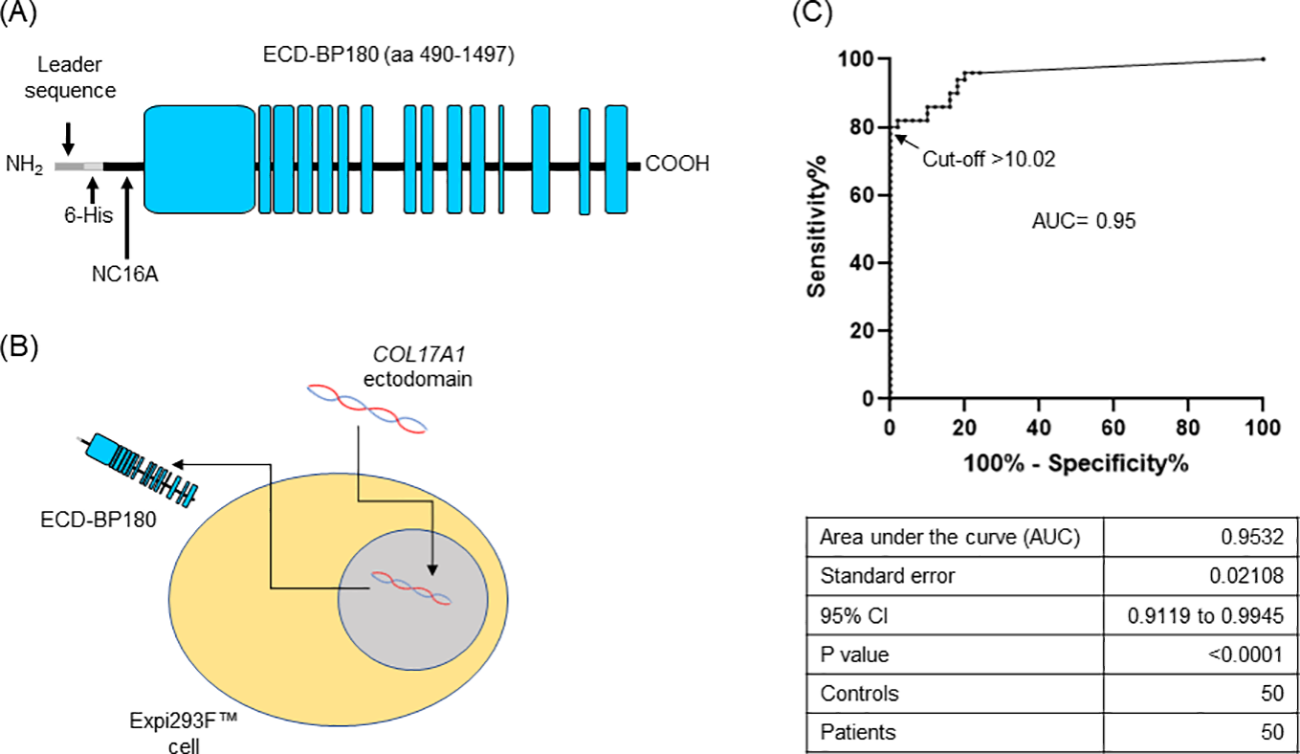
Setting up of BP180-ectodomain ELISA. **(A)** Schematic representation of the extracellular portion of human BP180 (ECD-BP180), spanning amino acids 490–1,497. Leader sequence for secretion, 6-His tag, and NC16A region are indicated. Black and light blue boxes refer to non-collagenous and collagenous protein domains, respectively. **(B)** ECD-BP180 production by transient transfection of Expi293™ cells is depicted. **(C)** Area under the curve (AUC) and cutoff value were calculated with 50 bullous pemphigoid patients and 50 normal donors.

ECD-BP180, comprising the amino acid (AA) sequence from 490 to 1,497, was produced in the eukaryotic system as a secreted protein (**Figure 1A**). A total of 87 nucleotide residues, coding for the Kozak consensus sequence, secretory signal peptide (Ig*k*), and 6×Histidine tag, were added at the 5′-end. The synthesized product was cloned in pcDNA3.1/Zeo vector by using *HindIII* and *NotI* cutting sites [GenScript Biotech (Netherlands) B.V., Leiden, Netherlands]. Expi293F™ cells (Thermo Fisher Scientific, Waltham, MA, USA) were transiently transfected with the resulting ECD-BP180 expressing plasmid and the culture medium was collected (**Figure 1B**).

### Enzyme-linked immunosorbent assays

We set up an ELISA by coating on microtiter plates (Thermo Fisher Scientific, Waltham, MA, USA) 50 µL of supernatant from ECD-BP180 transfected cells, or an equal volume from a control medium (mock), in 100 µL/well of 50 mM bicarbonate buffer (pH 9.6), overnight at 4°C. After washing twice in 0.1% (v/v) Tween20–Tris-buffered saline (T-TBS), wells were blocked with 1% BSA-T-TBS for 2 h at room temperature (RT) and subsequently incubated with 1:50 diluted sera in blocking buffer for 1 h with gentle shaking. Plates were washed six times with washing solution, and a 10,000-fold dilution of horseradish peroxidase (HRP)-conjugated rabbit anti-human IgG (Jackson ImmunoResearch Europe LTD, Ely, UK) was added (1 h with gentle shaking). After washing six times, color development was achieved by using 100 µL/well of 3,3′,5,5′-tetramethylbenzidine for 15 min; the reaction was stopped with 2N H_2_SO_4_ (50 µL/well) and optical density (OD) was read at 450 nm, with the correlation wavelength set at 655 nm using a microplate reader (Bio-Rad Hercules, CA, USA). Positive and negative control sera were included in each assay to minimize plate-to-plate variability. For each serum, a pemphigoid index value (PIV) was calculated as previously described (6). A receiver operating characteristic (ROC) curve of PIV values from 50 BP and 50 normal donor (ND) sera samples was calculated, and a cutoff point, based on the maximization of the Youden index (*J* = sensitivity + specificity − 1), was set at 10.02 PIV. The observed diagnostic specificity and sensitivity were 100% and 80%, respectively (**Figure 1C**).

To detect IgG reactivity against two ECD-BP180 epitopes, E-1080 and E-1331, spanning AA residues 1,080–1,107 and 1,331–1,404, respectively, we utilized the ELISA system previously described (26).

Commercial ELISA kits were used to assess the reactivity against BP180, BP230 (MBL, Nagoya, Japan), and collagen VII (Euroimmun AG Lübeck, Germany).

### Immunoblotting analysis

Supernatant medium from Expi293F™ transfected cells was separated in 6% SDS-PAGE under reducing conditions and electroblotted on PVDF membrane (Merck KGaA, Darmstadt, Germany). Membrane strips, probed with 20-fold diluted serum samples, were subsequently incubated with 1,000-fold diluted alkaline phosphatase (AP)-conjugated goat anti-human IgA (Merck KGaA, Darmstadt, Germany). The reactivity of MMP sera to laminin 332 was investigated by immunoblotting (IB) as previously described (16).

### Statistical analysis

Fisher’s exact test was used to assess the statistical significance of differences between mucosal involvement and epitope targeted in MMP and BP patients (*p* ≤ 0.05). To compare IgG titers, a non-parametric, unpaired Mann–Whitney test was employed. Data from descriptive analyses were reported as mean ± standard deviations and percentage.

## Results

### Characterization of IgG and IgA humoral response in bullous pemphigoid patients

BP patients presented a male/female ratio of 1.05 with a mean age of 76.8 years ( ± 12.3) (**Table 1**). Mucosal involvement was present in 24% of patients, and in 13%, the oral mucosa was exclusively affected (**Table 1**). To characterize their humoral response, the commercially available and in-house ELISAs as well as IB on recombinant proteins were employed. Of 90 BP sera, 79 (88%) possessed circulating IgG to BP180-NC16A, 48/90 (53%) to BP230, and 32 and 35 of 89 (36% and 39%) were positive for E-1080 and E-1331, respectively. Positivity to ECD-BP180 was found in 71/90 BP sera (79%) (**Table 1**). Of note, reactivity with one or more antigens was demonstrated in all 90 BP patients (100%) when BP180-NC16A, BP230, E-1080, and ECD-BP180 ELISAs were used (**Table 1**).

**Figure 2:**
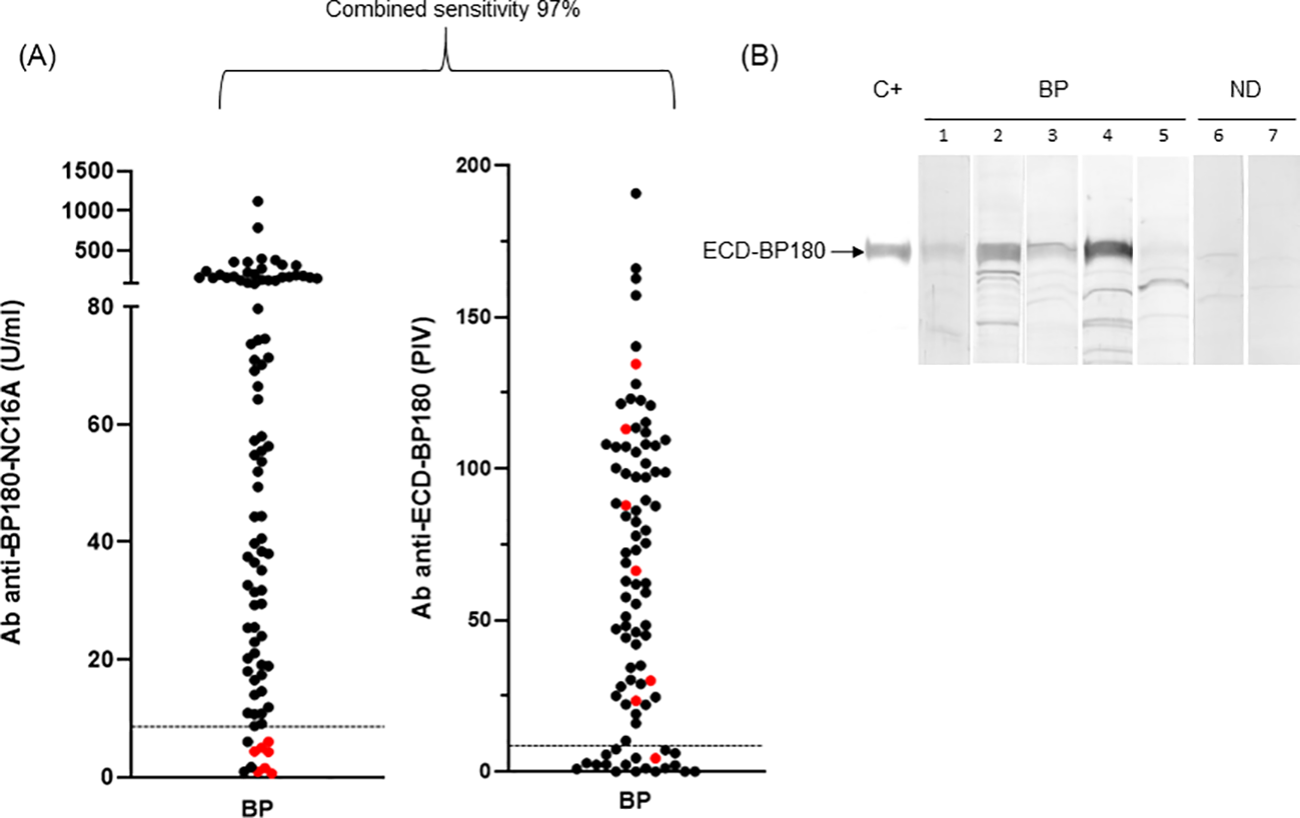
ELISA and immunoblotting reactivity to BP180 of bullous pemphigoid sera. **(A)** Scatter plot representation of IgG reactivity of bullous pemphigoid (BP) sera tested in BP180-NC16A and ECD-BP180 ELISAs. Red dots represent sera negative for both NC16A and BP230. The cutoff value is indicated by the dashed black line; PIV, pemphigoid index value, calculated as reported in Materials and Methods. **(B)** Immunoblotting filters showing IgA reactivity of BP and normal donor (ND) sera against recombinant ECD-BP180. Representative BP sera (1, 2, 3, and 4 positive and 5 negative), two ND (6 and 7 negative), and positive control serum (C+) are shown in the figure.

The combined sensitivity of commercial BP180 and BP230 ELISAs increased from 92% to 97% when the ECD-BP180 assay replaced the BP230 ELISA kit (**Figure 2A**). In particular, six of seven BP sera that showed no reactivity against BP180-NC16A and BP230 antigens (double negative) became positive via ECD-BP180 ELISA (**Figure 2A**). Interestingly, among seven double-negative BP sera, five were gliptin-associated BP, and all were positive via ECD-BP180 ELISA.

As for IgA humoral response, more than half (30/55, 55%) of analyzed BP reacted with ECD-BP180 by IB (**Figure 2B**). No difference in the detection of IgA and IgG autoAbs to ECD-BP180 between BP with and without mucosal involvement was observed (IgA: 57.9% vs. 57.6%, *p* > 0.999; IgG: 80% vs. 83%, *p* = 0.750).

### Characterization of IgG and IgA humoral response in mucous membrane pemphigoid patients

**Figure 3:**
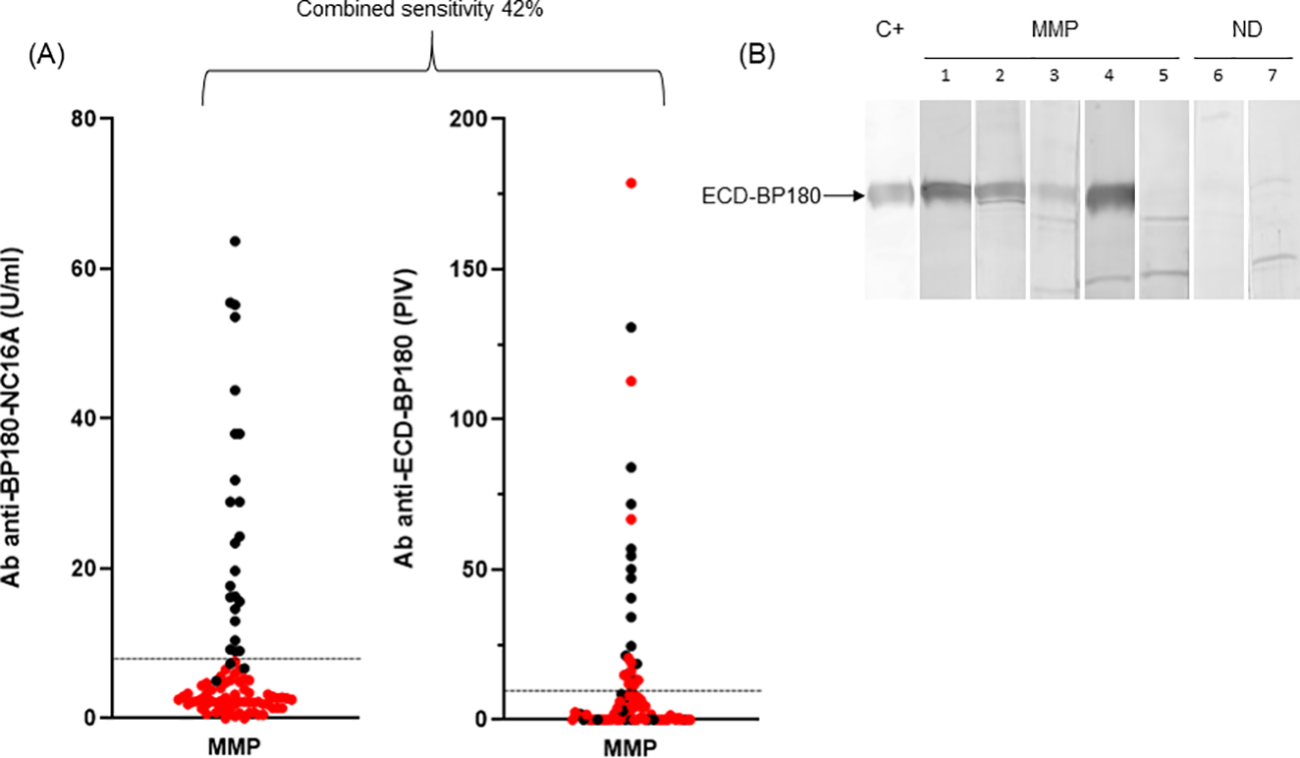
ELISA and immunoblotting reactivity to BP180 of mucous membrane pemphigoid sera. **(A)** Scatter plot representation of IgG reactivity of mucous membrane pemphigoid (MMP) sera tested in BP180-NC16A and ECD-BP180 ELISAs. Red dots represent NC16A and BP230 negative sera. The cutoff value is indicated by the dashed black line; PIV, pemphigoid index value, calculated as reported in Materials and Methods. **(B)** Immunoblotting filters showing IgA reactivity of MMP and normal donor (ND) sera against recombinant ECD-BP180. Five representative MMP sera (1, 2, 3, and 4 positive and 5 negative), two ND (6 and 7 negative), and positive control serum (C+) are shown in the figure.

MMP patients presented a male/female ratio of 0.51 with a mean age of 67.5 ( ± 13.7) (**Table 2**). In the cohort of 90 MMP, 25 (28%) were positive to BP180-NC16A and 10 (11%) were positive to BP230. Moreover, 3% (3/90) and 7% (6/90) exceeded the cutoff value with E-1080 and E-1331 ELISAs, respectively (**Table 2**). Of note, 28/90 (31%) MMP sera reacted with ECD-BP180 in IgG ELISA (**Table 2**). As occurred for BP, the combined sensitivity of BP180-NC16A and ECD-BP180 ELISAs was greater compared to BP180-NC16A and BP230 ELISAs (42% and 31%, respectively) (**Figure 3A**). Interestingly, 12/62 (19%) MMP double-negative sera became positive by using ECD-BP180 recombinant protein (**Figure 3A**). As for IgA circulating autoAbs, IB studies determined positivity to ECD-BP180 in 30/76 (40%) analyzed sera (**Figure 3B**); notably, 50% of these were not detected by IgG ELISAs. Overall, IgA reactivity to ECD-BP180 was more frequent than IgG in MMP patients. Of the 75 MMP patients analyzed, 11 (15%) possessed IgG to laminin 332 by IB, of which only two stained the dermal side of salt-split human skin (**Table 2**). As for collagen VII, 3/68 (5%) MMP patients analyzed were positive by ELISA and none were dermal side positive by IIF (**Table 2**).

The combined sensitivity reached 83% (75/90) when all described immunological assays, along with IgG and IgA IIF on salt-split skin, were performed, except for E-1331 ELISA whose reactivity did not improve the global sensitivity (**Table 2**).

### Midportions of BP180 and BP230 were less bound by autoantibodies in MMP compared to BP

**Figure 4:**
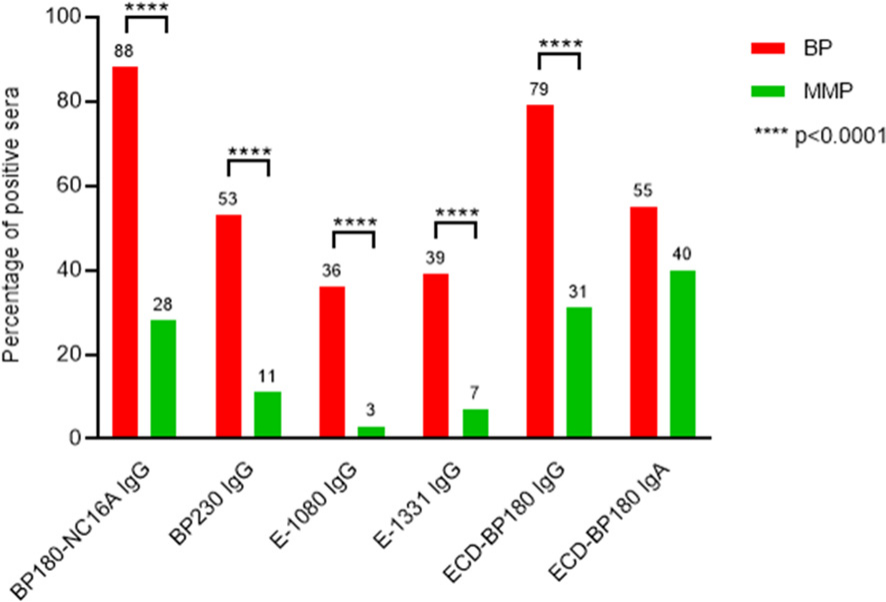
MMP patients’ sera show a global reduced reactivity to BP180 and BP230 in comparison with BP. The graph displays the comparison of immunological reactivity between bullous pemphigoid (BP) and mucous membrane pemphigoid (MMP) sera with BP230 and BP180 antigens/epitopes. E-1080, midportion of the extracellular domain of BP180, spanning amino acids (AA) 1,080–1,107; E-1331, C-terminal region of the extracellular domain of BP180, spanning AA 1,331–1,404; ECD-BP180, ectodomain region of BP180 covering the 490–1,497 AA residues; *p*-value was evaluated with Fisher’s exact test.

The characterization of the MMP epitope profile showed a global decreased reactivity in comparison with BP (**Figure 4**, **Tables 1**, **2**). In fact, in MMP patients, BP180 and BP230 showed a significant reduction of frequency of IgG autoAbs (*p* < 0.0001) (**Figure 4**) and of IgG mean titers (*p* < 0.0001 for both BP180 and BP230), except for E-1331 (**Tables 1**, **2**). However, the above-mentioned reductions mostly affected BP230 and E-1080. Specifically, the frequency of IgG positivity to BP230 and E-1080 in MMP compared to BP was 11% and 3% vs. 53% and 36%, respectively (*p* < 0.0001) (**Figure 4**, **Tables 1**, **2**). Of note, although rare, reactivity against these two antigens was mainly present in MMP with exclusive oral involvement (**Table 3**). IgG mean titers to BP230 and E-1080 were also reduced in MMP compared to BP (26 vs. 64 U/mL, *p* = 0.0053 and 46 vs. 101 PIV, *p* = 0.9091, respectively) (**Tables 1**, **2**). Notably, the sharp difference measured in E-1080 titers did not reach a statistically significant *p*-value due to the few E-1080 positive cases in MMP.

**Table 3:** Reactivity of MMP patients’ sera with different mucosal sites involved.

	Oral	Ocular	One or more sites + cutaneous	Oral vs. ocular	Oral vs. one or more sites + cutaneous	Ocular vs. one or more sites + cutaneous
*n*/*N* (%)				*p*-value		
**IgG anti-** **BP180-NC16A**	14/42 (33.3)	1/13 (7.7)	8/28 * (28.6)	0.0863	0.7947	0.2283
**IgG anti-** **BP230**	5/42 (11.9)	2/13 (15.4)	2/28 (7.1)	0.6639	0.6941	0.5795
**IgG anti-** **E-1080**	3/42 (7.1)	0/13 (0)	0/28 (0)	0.5624	0.1444	>0.999
**IgG anti-** **E-1331**	3/42 (7.1)	0/13 (0)	1/28 (3.6)	0.5624	0.6415	>0.999
**IgG anti-** **ECD-BP180**	14/42 (33.3)	0/13 (0)	9/28 (32.1)	**0.0245**	>0.999	**0.0377**
**IgA anti-** **ECD-BP180**	14/34 (41.2)	4/12 (33.3)	11/27 (40.7)	0.7393	>0.999	0.7342

*A total of 28 patients with one or more mucosal sites and skin affected: 5 oral, 4 ocular, and 19 multiple mucosal sites. E-1080 and E-1331, midportion (AA 1,080–1,107) and C-terminal region (AA 1,331–1,404) of the extracellular domain of BP180; ECD-BP180, ectodomain of BP180 (AA 490–1,497); *n*/*N*, positive/total. Statistically significant *p* values evaluated with Fisher’s exact test are in bold.

### MMP patients with exclusive oral or cutaneous involvement react to BP180 by IgG more than MMP patients with ocular involvement

In our cohort of MMP patients, 64/86 (74%) presented oral involvement alone or together with other cutaneous or mucosal sites. Out of 86 MMP patients, nearly half (*n* = 42, 49%) displayed an exclusive oral involvement and 13 (15%) showed only ocular lesions, and in 22 patients (26%), multiple mucosal sites were affected. Interestingly, almost one-third of MMP patients (28/86) showed both cutaneous and mucosal involvement. The frequency of IgG reactivity to BP180-NC16A and ECD-BP180 was higher in the oral than in the ocular variant (*p* = 0.0863 and *p* = 0.0245, respectively) (**Table 3**). Similar results were obtained for 28 MMP patients with concurrent cutaneous and mucosal involvement affecting oral or ocular or multiple sites in comparison with the exclusively ocular variant (BP180-NC16A: *p* = 0.2283 and ECD-BP180: *p* = 0.0377, respectively) (**Table 3**). Of note, MMP patients with both oral and cutaneous involvement showed the highest reactivity to BP180-NC16A and ECD-BP180 (60% and 80%, respectively) (**Supplementary Table S1**).

### Pure ocular MMP patients mainly react to ECD-BP180 by IgA

We observed a prevalent IgA reactivity against ECD-BP180 regardless of the mucosal site involved (**Table 3**). Of note, one-third of ocular MMP (4/12) showed IgA positivity to ECD-BP180 by IB (**Table 3**). In addition, considering 17 MMP patients with ocular involvement (13 exclusively ocular and 4 with skin and ocular lesions), 5 (29%) reacted with ECD-BP180 by IgA, while 2 (12%) reacted with BP180-NC16A and/or BP230, and 10 (59%) were IgG and IgA negative to all antigens/epitopes tested (**Table 3**; **Supplementary Table S1**).

## Discussion

The differential clinical diagnosis between BP and MMP was based on the time of appearance and the extension of cutaneous and/or mucosal lesions. However, in some patients with concomitant mucosal and cutaneous involvement, especially if the temporal appearance of mucosal lesions is difficult to recall and/or because of the evolution of cutaneous/mucosal involvement, it is more difficult to discern the most prevalent feature and the differential diagnosis can be challenging. In addition, the non-bullous forms of BP may complicate the diagnosis (27). Distinguishing between these pemphigoid forms can be crucial for defining a correct therapy and prognosis. In fact, the therapeutic approach may be different between BP and MMP; for example, although topical steroids are effective in BP, they may not work well in some types of MMP (28). Moreover, in selected cases of MMP, the prognosis could be more severe than BP, especially when mucosal involvement evolves towards ocular lesions that could result in reduced vision or even corneal perforation (24).

In the present study, the comparison of immunological reactivity profiles of the largest published cohorts of BP and MMP (8, 29–32), except for the study from Yasukochi et al. investigating only anti-BP180 reactivity in MMP (33), has been performed. As expected, MMP sera showed a global decreased reactivity in frequency and titers to BP180 and BP230. Of note, BP230 (53% vs. 11%; *p* < 0.0001) and E-1080 (36% vs. 3%; *p* < 0.0001) were mostly affected. Likewise, a lower positivity was obtained with E-1080 and/or E-1331 ELISAs (MMP 9% vs. BP 57%; *p* < 0.0001). We think that these results can be useful for the diagnosis of doubtful cases. In fact, considering that 97% of BP recognizes NC16A and/or 1080 and/or BP230 and that 68% of MMP is instead negative for all these antigens/epitopes, if a case is negative for all these antigens/epitopes, it is almost certainly an MMP (within a 3% error). Accordingly, among the doubtful cases when it comes to MMP, it will be correctly diagnosed in two out of three cases.

Several studies postulated the involvement of epitope spreading phenomenon in MMP (34); however, the dynamics of the immune response was finely described only in BP. Specifically, the BP immune response initially targets NC16A and then spreads to intracellular epitopes/antigens, e.g., BP230 and other BP180 epitopes (35), with tissue damage and inflammation being at the base of epitope spreading phenomena (36). Similarly, a mechanism of epitope spreading could be supposed for MMP, but further extensive studies are required to prove it. Interestingly, in animal models and humans, it was demonstrated that wound healing was faster and with less scarring in oral mucosa compared to skin (37–39). These differences could depend on growth factor production, stem cell levels, and cellular proliferation capacity (40–42). In addition, BP180 expression was markedly higher in oral than in skin keratinocytes and correlated with higher adhesion strength, potentially affecting the wound healing process (43). Thus, the diminished frequency of BP230 and E-1080 reactivity in MMP could depend on, at least in oral mucosa, a faster wound healing process, limiting the chronicity of tissue damage and the activation and recruitment of autoreactive lymphocytes involved in epitope spreading phenomena.

The frequency and titer reduction of IgG reactivity to BP180 and BP230 could also be due to isotype switching from IgG to IgA. However, IgA is very permeable to the epithelium and tends to be shed into the lumen, accounting for the apparent negativity or titer reduction detected in serum. In line with these observations, although IgA is a typical component of the mucosal immune system, circulating anti-ECD-BP180 IgA is not always related to the mucosal phenotype. In fact, they are (i) more present in BP (55%) than in MMP (40%) and (ii) not associated with mucosal involvement in BP. In accordance with our data, several other reports do not show a definitive correlation between the presence of IgA reactivity and mucosal involvement (6, 8, 44, 45).

Of note, the present study demonstrates that the MMP autoAb profiles are partially associated with different mucosal or cutaneous sites affected. In particular, IgG reactivity to BP180 was associated with the involvement of oral mucosa and increased when the skin was concurrently affected (**Table 3**; **Supplementary Table S1**), while it was rare in ocular MMP, where BP180 IgA reactivity was relevant instead. In line with literature data (46), our results show that MMP patients with ocular involvement rarely reacted to BP180 by IgG in contrast with patients with oral and/or cutaneous involvement. Thus, it could be hypothesized that an oral and/or cutaneous MMP, positive to BP180, hardly evolves to ocular MMP, suggesting a favorable prognosis outcome.

These differences could depend on structural characteristics making BP180 epitopes more detectable in the skin and oral mucosa compared to the ocular site and/or on the prevalence of IgA in the ocular area. Furthermore, in almost 60% of MMP patients with exclusively ocular or cutaneous and ocular involvement, the antigen was not identified, suggesting the presence of autoAbs against other hitherto unknown antigens, such as the integrin beta 4 subunit, referred to as the almost exclusive antigen of ocular MMP in some studies (47). Recently, diagnostic improvements for ocular MMP were considered necessary (24, 48). In fact, at diagnosis, several patients have severe conjunctival inflammation, advanced cicatrizing disease, and symblepharon formation, suggesting a possible diagnostic delay (49, 50). In addition, several cases of ocular MMP cannot be confirmed by DIF. Thus, to exclude other cicatrizing conjunctival disorders, this subset of ocular MMP requires more sensitive diagnostic assays. In the present study, IgA-IB on ECD-BP180 improves the sensitivity of IgG ELISAs in MMP patients with ocular involvement supporting a difficult diagnosis in almost one-third of cases. In addition, in the present study, the combined ECD-BP180 and BP180-NC16A ELISAs showed a higher sensitivity than the BP180-NC16A/BP230 combination in both BP and MMP sera, improving the diagnostic performance of commercially available assays.

The limitations of our study mainly concern missing data on reactivity to other antigens such as the α6 and β4 integrin subunits, as well as on disease severity and treatment. It would be valuable to shed light on sera reactivity against integrins, not only for the subset negative to BP180 and BP230, but also for the positive one. In this case, a mutually exclusive or a concurrent autoAbs reactivity could be revealed. In conclusion, our findings (i) highlight the importance of BP230 and E-1080 ELISA to distinguish between BP and MMP in challenging cases, (ii) suggest BP180 positivity as an MMP prognostic marker associated with the absence of ocular involvement, and (iii) improve the diagnostic performance of commercial ELISAs employing the detection of IgG and IgA to ECD-BP180.

Future longitudinal studies on ocular MMP patients are advisable to strengthen the prognostic role of ECD-BP180 IgA reactivity.

## Data Availability

The original contributions presented in the study are included in the article/**Supplementary Material**. Further inquiries can be directed to the corresponding author.
