# Supplementary material for: Bullous pemphigoid and mucous membrane pemphigoid humoral responses differ in reactivity towards BP180 midportion and BP230
Source: Front Immunol. 2024 Nov 29;15:1494294. doi: 10.3389/fimmu.2024.1494294 (PMC11638032; doi:10.3389/fimmu.2024.1494294)
Supplement: Supplementary file 1 [file Table1.docx]

| **Supplementary Table S1. Reactivity of MMP patients’ sera with different mucosal sites and cutaneous involvement** | | | |
| --- | --- | --- | --- |
| **oral and ocular and multiple sites**  **cutaneous cutaneous and cutaneous** | | | |
|  | ***n/N***  **(%)** | ***n/N***  **(%)** | ***n/N***  **(%)** |
| **IgG anti-**  **BP180-NC16A** | 3/5  (60.0) | 0/4  (0) | 5/19  (26.3) |
| **IgG anti-BP230** | 1/5  (20.0) | 0/4  (0) | 1/19  (7.1) |
| **IgG anti-E-1080** | 0/5  (0) | 0/4  (0) | 0/19  (0) |
| **IgG anti-E-1331** | 1/5  (20.0) | 0/4  (0) | 0/19  (0) |
| **IgG anti-**  **ECD-BP180** | 4/5  (80.0) | 0/4  (0) | 5/19  (26.3) |
| **IgA anti-**  **ECD-BP180** | 4/5  (80.0) | 1/4  (25.0) | 6/18  (33.3) |
| E-1080 and E-1331, mid-portion (AA 1,080-1,107) and C-terminal region (AA 1,331-1,404) of the extracellular domain of BP180; ECD-BP180, ectodomain of BP180 (AA 490-1,497); IIF-sss, indirect immunofluorescence on salt-split skin. | | | |
